# Identification of a long non-coding RNA NR_026689 associated with lung carcinogenesis induced by NNK

**DOI:** 10.18632/oncotarget.7475

**Published:** 2016-02-18

**Authors:** Jianjun Wu, Xun Li, Yiqin Xu, Ti Yang, Qiaoyuan Yang, Chengfeng Yang, Yiguo Jiang

**Affiliations:** ^1^ State Key Laboratory of Respiratory Disease, Institute for Chemical Carcinogenesis, Guangzhou Medical University, Guangzhou 511436, People's Republic of China; ^2^ Department of Physiology and Center for Integrative Toxicology, Michigan State University, East Lansing, MI 48824, USA

**Keywords:** lncRNA, NR_026689, lung carcinogenesis, NNK

## Abstract

Long non-coding RNAs (lncRNA) are thought to be important epigenetic regulators involved in the development of a variety of cancers. Alterations in lncRNA expression are associated with exposure to chemical carcinogens. However, it is still unclear whether lncRNA expression during lung carcinogenesis is induced by chemical carcinogens. In this study, using NNK-induced rat lung cancer model established by our previous study, we determined the lncRNA expression profiles, and an alteration in lncRNA expression was observed in lung cancer tissues and blood in the NNK treatment group. Using quantitative reverse-transcription PCR (qRT-PCR), five differentially expressed lncRNAs were further detected and validated. We identified a novel lncRNA, NR_026689, which showed increased expression in lung cancer tissues induced by NNK and the alteration of lncRNA NR_026689 was specifically observed in lung tissue. The level of NR_026689 was determined and significantly increased in rat whole blood at the 10th and 20th week after NNK treatment to evaluate it as a potential early marker for lung cancer. Together, these findings suggest that lncRNA NR_026689 may be a potential early biomarker for lung cancer and is associated with lung carcinogenesis induced by NNK.

## INTRODUCTION

Lung cancer is a leading cause of all cancer deaths worldwide. It is known that cigarette smoke and air pollution are the main risk factors for lung cancer [1, 2]. Despite significant efforts to improve treatment options for patients with lung cancer, the 5-year survival rate for lung cancer patients is still only about 17% [3]. Due to lack of early diagnostic biomarker, most cases of lung cancer are diagnosed at an advanced stage. The discovery of diagnostic biomarkers for early stage lung cancer would potentially significantly improve the five-year survival rate of lung cancer patients [4].

Long non-coding RNAs (lncRNAs) are transcripts which are > 200 bp in length and do not encode protein. In the past, these lncRNAs were believed to be transcripts without a function. However, in the past decade, numerous studies have demonstrated that lncRNAs have distinct biological functions in development [5], apoptosis [6, 7], proliferation and differentiation [8], and play important roles in various human diseases, including cancer [9]. Recent studies have shown that abnormal expression of lncRNAs is associated with a variety of cancers [10], such as gastric cancer [11, 12], prostate cancer [13], hepatocellular carcinoma [14, 15], breast cancer [16] and lung cancer [17]. LncRNAs were also thought as potential biomarkers for a variety of cancers [18, 19].

Several previous studies reported that chemical carcinogens cause alterations in lncRNA expression. Bisphenol-A and diethylstilbestrol exposure induced alterations in the expression of the lncRNA, HOTAIR, in cultured human breast cancer cells (MCF7) as well as in the mammary glands of rats [20]. In workers occupationally exposed to benzene, the lncRNA, NR_045623 and NR_028291, were thought to be key genes associated with benzene hematotoxicity [21]. According to Leslie Recio, exposure to the chemical carcinogen, furan, can induce differential expression of lncRNAs in the liver tissue of female B6C3F1 mice [22]. Thai et al. identified a novel lncRNA, SCAL1, induced by cigarette smoke [23]. These studies indicate that chemical exposure can induce alterations in lncRNA expression.

4-(methylnitrosamino)-1-(3-pyridyl)-1-butanone (NNK) is a key compound in cigarette smoke which plays a major role in lung carcinogenesis [24]. Numerous studies have shown that NNK is a potent lung carcinogen in rats, mice, and hamsters. NNK-induced lung tumorigenesis exhibited marked organ specificity in male F344 rats [25, 26]. In our previous study [27], we successfully established an F344 rat model of lung carcinogenesis induced by NNK and collected whole blood at different stages of NNK-induced lung carcinogenesis and tissue samples at 95 weeks after NNK treatment. Silva et al. added NNK to normal human bronchial epithelial cells (NHBE) and identified 12 Long Stress-Induced Non-coding Transcripts [28]. To date, the alteration in expression of lncRNAs is unclear during NNK-induced lung carcinogenesis. In the present study, using the rat model, the differential expression profiles of lncRNA were analyzed using the Arraystar rat lncRNA array. lncRNA NR_026689 was identified as a potential biomarker for lung cancer and was associated with lung carcinogenesis induced by NNK.

## RESULTS

### Differential expression profiles of lncRNA in NNK-induced lung carcinogenesis

**Figure 1:**
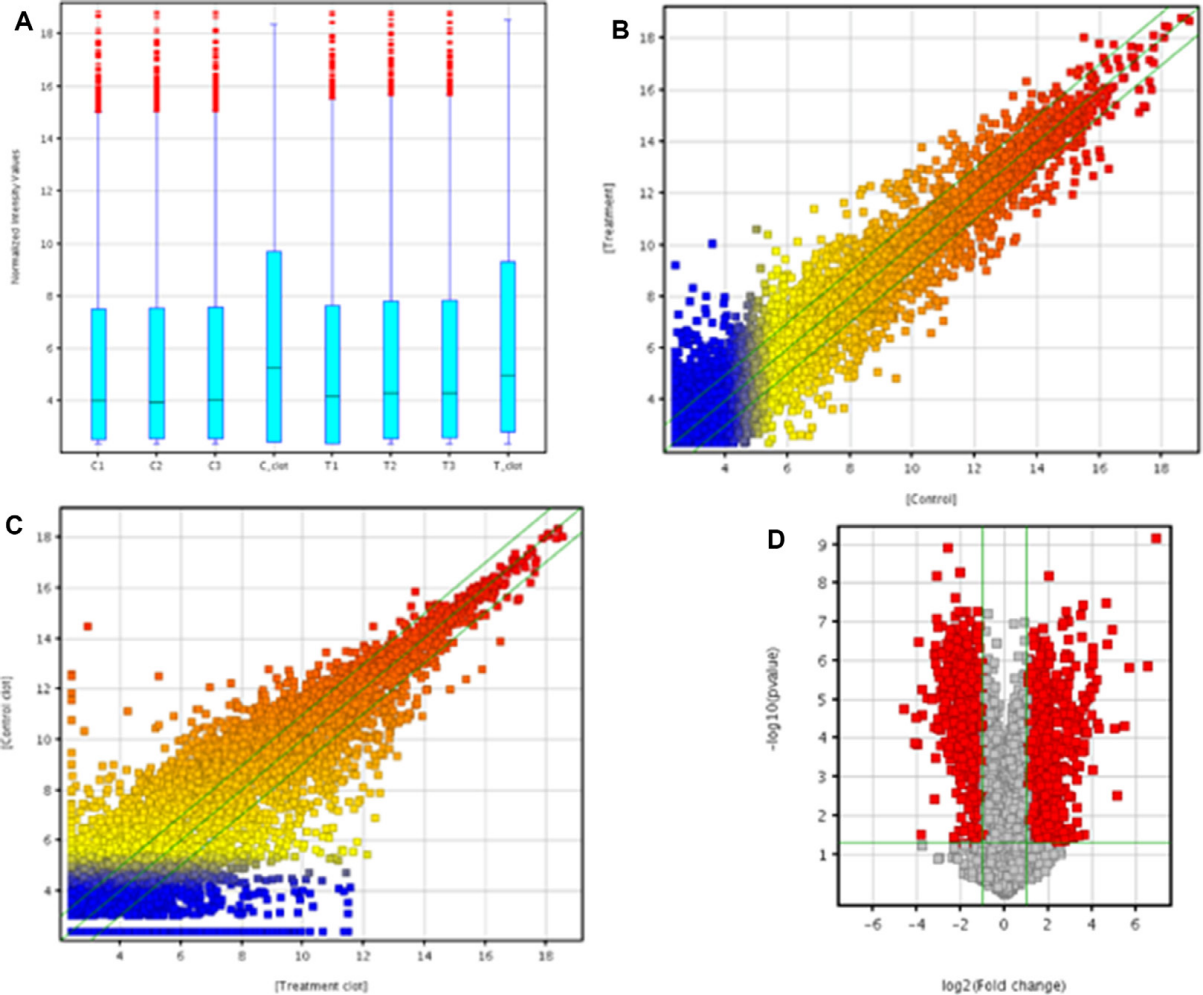
Microarray analysis of lncRNA expression profile The box plot is used for comparing the distributions of the intensities from all samples (**A**) After normalization, the distributions of log2-ratios among all tested samples are nearly the same. The Scatter-Plot is used for assessing lncRNA expression variation between lung tumor tissues and matched normal lung tissues of NNK treatment group (**B**) The Scatter-Plot is used for assessing lncRNA expression variation in blood samples between control and NNK treatment group (**C**) The values of X and Y axes in the Scatter-Plot are the averaged normalized signal values of the samples in each group (log2 scaled). The green lines are 2-fold change lines. The lncRNAs above the top green line and below the bottom green line indicated more than 2 fold change of lncRNAs between the two compared groups. Volcano Plot analysis of the microarray data on the differentially expressed lncRNA in lung tumor tissues vs. matched normal lung tissues of NNK treatment group (**D**) The vertical lines correspond to 2.0- fold up and down regulation while the horizontal line represents a *p* value of 0.05. The red point in the plot represents the differentially expressed lncRNAs with statistical significance. Statistical significance was defined as fold change ≥ 2.0 and *p* value ≤ 0.05 between lung tumor tissues and matched normal lung tissues of NNK treatment group.

**Figure 2:**
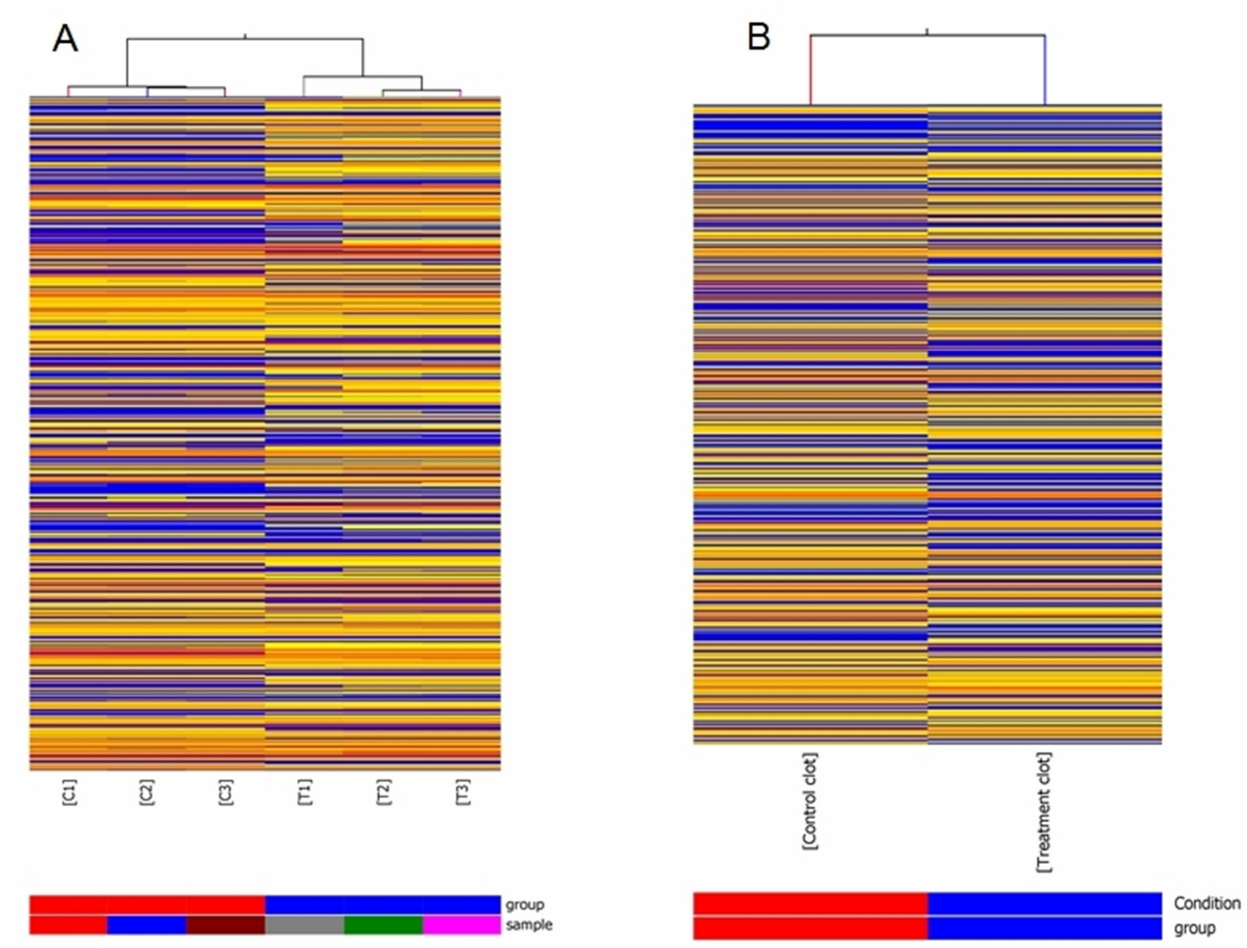
Heat map presentation of expression profile of lncRNA The heat maps show that the correlation for lncRNAs that were statistically differentially expressed in lung tumor tissues vs. matched normal lung tissues of NNK treatment group at the 95th week (**A**). T1-3: three independent lung tumor samples from NNK treatment group respectively. C1-3: three matched normal lung tissue samples from NNK treatment group, respectively. The heat maps for differentially expressed lncRNAs in blood samples between NNK treatment group and control group at the 95th week (**B**). Control clot and Treatment clot respectively represent blood samples from control group and NNK treatment group. Each column represents a separate sample and each row represents a single lncRNA. “Red” indicates high relative expression, and “blue” indicates low relative expression.

In order to assess the alteration in lncRNA expression in rat lung carcinogenesis induced by NNK, we selected 3 pairs of rat lung tumor samples and matched normal lung tissues and 2 blood samples from the control and NNK treatment group in the 95th week, and determined the lncRNA expression profiles using Arraystar rat lncRNA microarray. After normalization for raw signal intensities in Quantile method by GeneSpring GX v11.5, and filtering for low intensity lncRNAs, the quality of lncRNA data was assessed by box plot and scatter plot. As shown in Figure 1A, after normalization, the distributions of intensities in all samples were almost the same. The scatter-plot was then used to assess the variation in lncRNA expression between the two compared arrays (Figure 1B and 1C). To identify differentially expressed lncRNAs which were significantly different, we performed Volcano Plot filtering between the compared groups (the filtering threshold was a fold change ≥ 2 and a *p* value ≤ 0.05). According to these screening factors, the differential expression profiles between lung tumor tissues and matched normal lung tissues are shown in Figure 1D. Compared with normal lung tissues, 757 lncRNAs were significantly up-regulated and 519 lncRNAs were significantly down-regulated in lung tumor tissues induced by NNK. The lncRNA differential expression profiles in blood samples between the control group and NNK treatment group indicated that 2130 lncRNAs and 1220 lncRNAs were up-regulated and down-regulated in the NNK treatment group, respectively. Hierarchical clustering was then performed based on differentially expressed lncRNAs in lung tissues and blood samples. The results showed the relationships and distinguishable lncRNA expression profiling among the samples. As shown in Figure 2A, hierarchical clustering distinguished between normal lung tissues and lung tumor samples. Similarly, hierarchical clustering also distinguished between blood samples from the control and NNK treatment group (Figure 2B). Details of the lncRNA microarray data in this study have been deposited in NCBI's Gene Expression Omnibus and are accessible through GEO Series accession number GSE68967 (https://www.ncbi.nlm.nih.gov/geo/query/acc.cgi?acc=GSE68967).

### Further screening and validation of differential expression of lncRNAs by qRT-PCR

**Figure 3:**
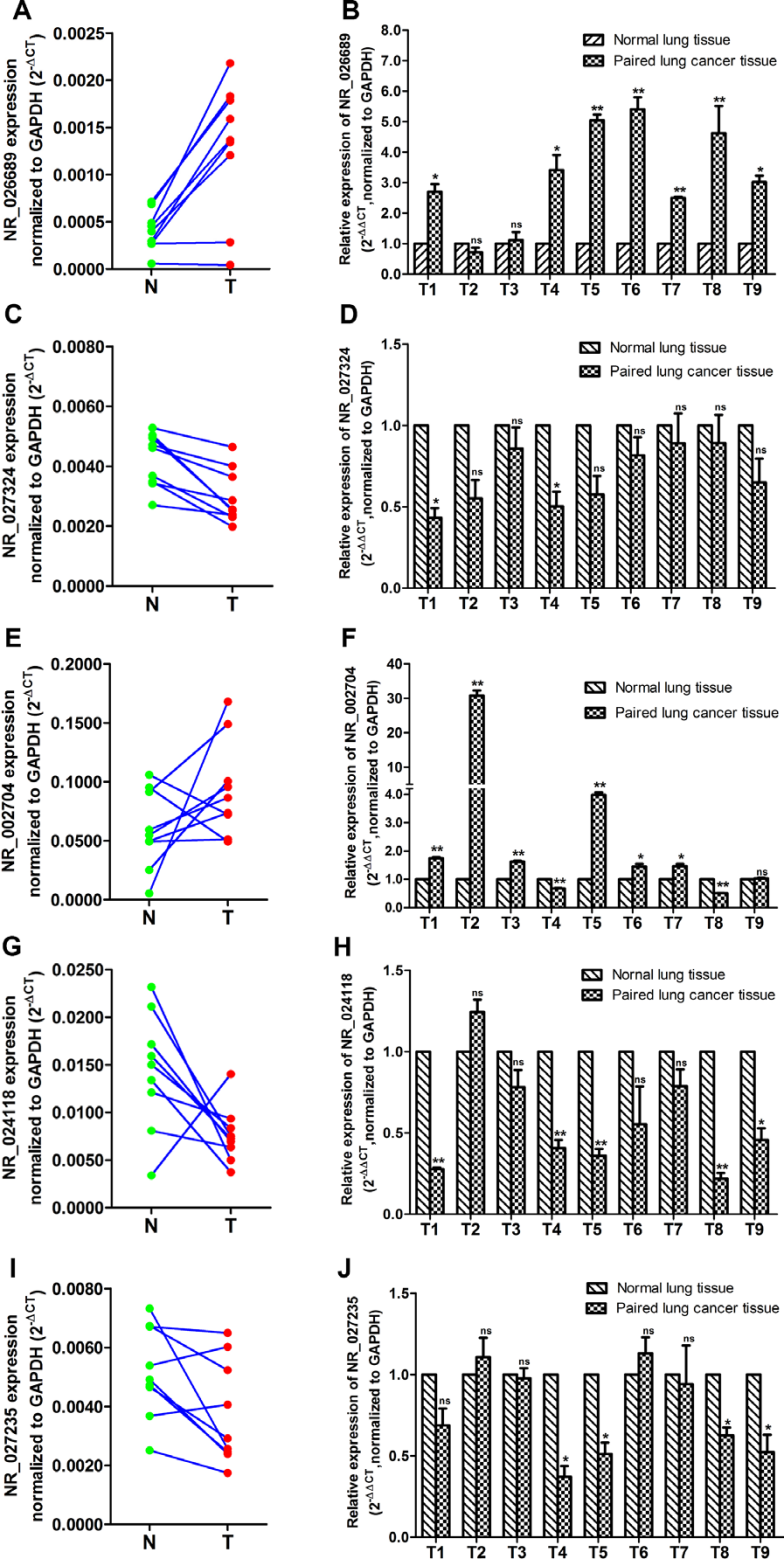
Identification of candidate lncRNAs by qRT-PCR analysis in rat lung tumor tissues vs. paired normal lung tissues from the NNK treatment group at the 95th week The expression level of lncRNA in 9 paired lung tissues was determined for NR_026689 (**A**) and (**B**), NR_027324 (**C**) and (**D**), NR_002704 (**E**) and (**F**), NR-024118 (**G**) and (**H**), and NR_027235 (**I**) and (**J**). T denotes the rat lung tumor tissues and N represents the matched normal lung tissues in the NNK treatment group. The expression level of lncRNA was normalized to GAPDH. The paired Student's *t* test was used to ascertain the statistical significance of the differences between rat lung tumor tissues and paired normal lung tissues in the NNK treatment group. Data are presented as mean ± SEM for three independent experiments, *p < 0.05; **p < 0.01; ns: no significance.

According to microarray manufacturer's recommendation, we selected differential expressed lncRNAs derived from NCBI RefSeq_NR for further studies. Then, five differentially expressed lncRNAs (NR_026689, NR_027324, NR_002704, NR_024118 and NR_027235) were selected as candidate lncRNAs for further analysis by screening the lncRNA microarray data. The details of differential expression in these lncRNAs are shown in Table 1. LncRNA NR_026689, NR_027324 and NR_002704 were significantly up-regulated; lncRNA NR_024118 and NR_027235 were significantly down-regulated, respectively. In order to validate the results of lncRNA microarray, the five candidate lncRNAs were determined in nine paired rat lung tumor tissues and matched normal lung tissues from NNK-treated rats in the 95th week using qRT-PCR. These results are shown in Figure 3. Compared with normal lung tissues in the NNK treatment group, the level of lncRNA NR_026689 (Figure 3A and 3B) and NR_002704 (Figure 3E and 3F) expression was significantly increased in nine paired lung tumor tissues in the NNK treated group (paired *t*-test, *n* = 9). Of these nine paired samples, the expression of NR_026689 and NR_002704 was significantly up-regulated in seven and six paired lung tumor tissues (7/9 and 6/9), respectively. There was no significant difference in the level of lncRNA NR_027324 between normal lung tissues and paired lung tumor tissues (Figure 3C and 3D). The level of lncRNA NR_024118 (Figure 3G and 3H) and NR_027235 (Figure 3I and 3J) expression was significantly decreased in nine lung tumor tissues compared to paired normal lung tissues in the NNK treated group (paired *t*-test, *n* = 9). NR_024118 and NR_027235 were significantly up-regulated in 5/9 and 4/9, respectively. These data indicated that the qRT-PCR results for lncRNA NR_026689, NR_002704, NR_024118 and NR_027235 were consistent with those of lncRNA microarray. Of these four lncRNAs, the differential expression of NR_026689 was more significant than the others. Thus, lncRNA NR_026689 was selected as a candidate lncRNA for further study.

**Table 1:** Candidate lncRNAs for additional qRT-PCR in individual rats

LncRNA Name	Intensity of control	Intensity of treatment	Fold change	Regulation	*P*-Value	Chromosome maps
NR_026689	179.07612	1160.225	4.857965	up	3.78E–06	Chr12_random: 743385–743445
NR_027324	22.51848	75.77106633	2.608754	up	4.83E–04	Chr13:40636333–40636393
NR_002704	1182.3719	3009.056467	2.3254766	up	2.95E–05	Chr13:76595646–76596186
NR_024118	3386.4756	699.3427367	4.128733	down	1.40E–04	Chr20:4128070–4128130
NR_027235	45.174255	24.038046	2.050225	down	0.0147523	Chr4: 76723961–76724021

### Analysis of the sequence of NR_026689 by 5′RACE

**Figure 4:**
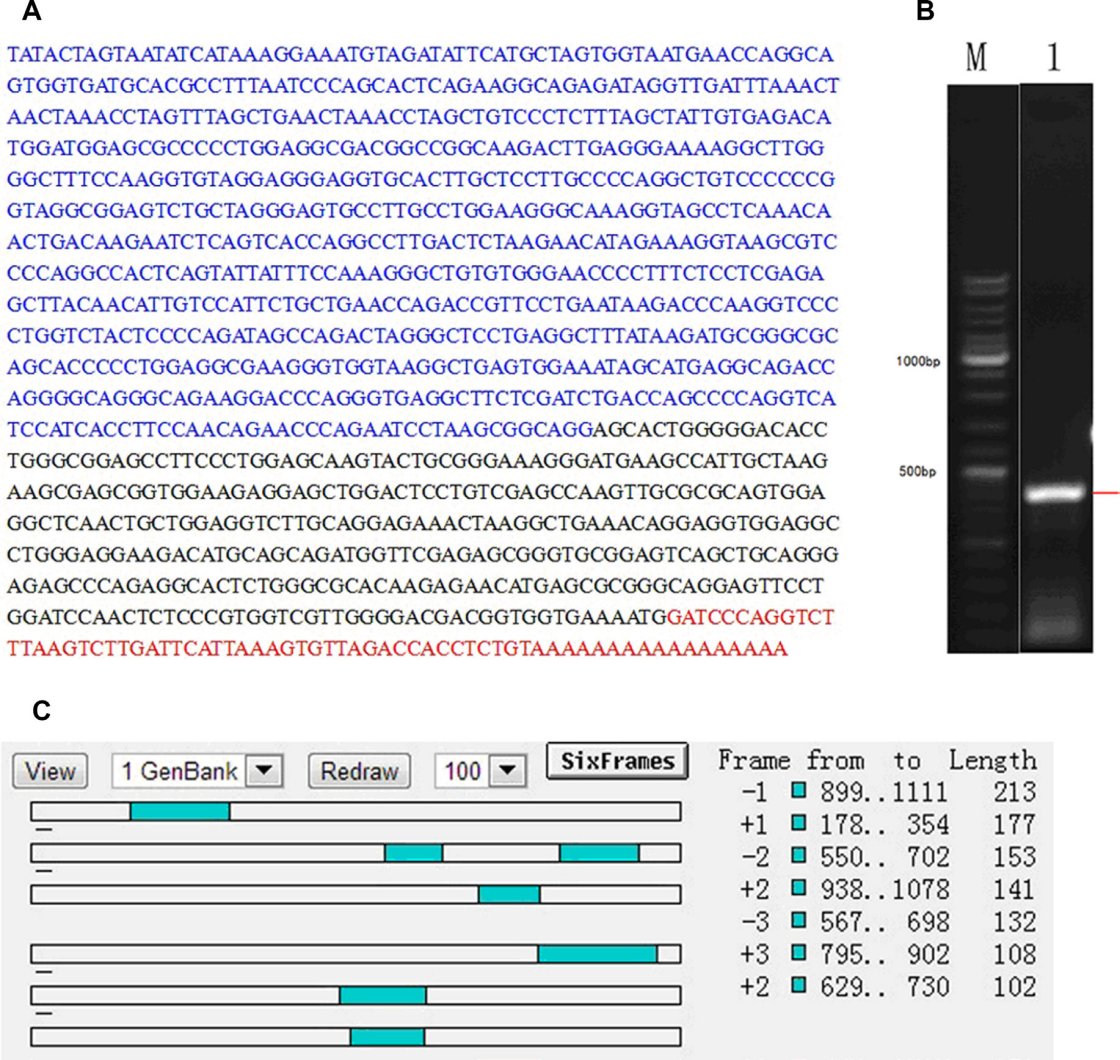
Results of 5′ RACE in NR_026689 (**A**) Nucleotide sequences of NR_026689, composed of a 5′ end sequence (blue), middle sequence (black) and 3′end sequence (red). (**B**) Agarose gel electrophoresis of nested PCR products from the 5′ RACE procedure. Lane M: 1 kb DNA Ladder; Lane 1: PCR product was amplified from the 5′end of NR_026689. (**C**) ORF prediction of NR_026689 sequence. No continuous ORFs are present in the NR_026689 sequence.

In order to understand the possibility of NR_026689 encoding protein, the nucleotide sequences and PCR products of NR_026689 were obtained by 5′RACE assay. The full-length sequences of NR_026689 and agarose gel electrophoresis of nested PCR products are presented in Figure 4A and 4B. Further analysis of the sequences was performed by ORF Finder and no continuous ORF was found. The longest ORF of NR_026689 was 213 bp (Figure 4C). The results indicate that NR_026689 is unlikely to be translated and conforms to the character of a lncRNA.

### The expression of lncRNA NR_026689 is associated with lung carcinogenesis induced by NNK

**Figure 5:**
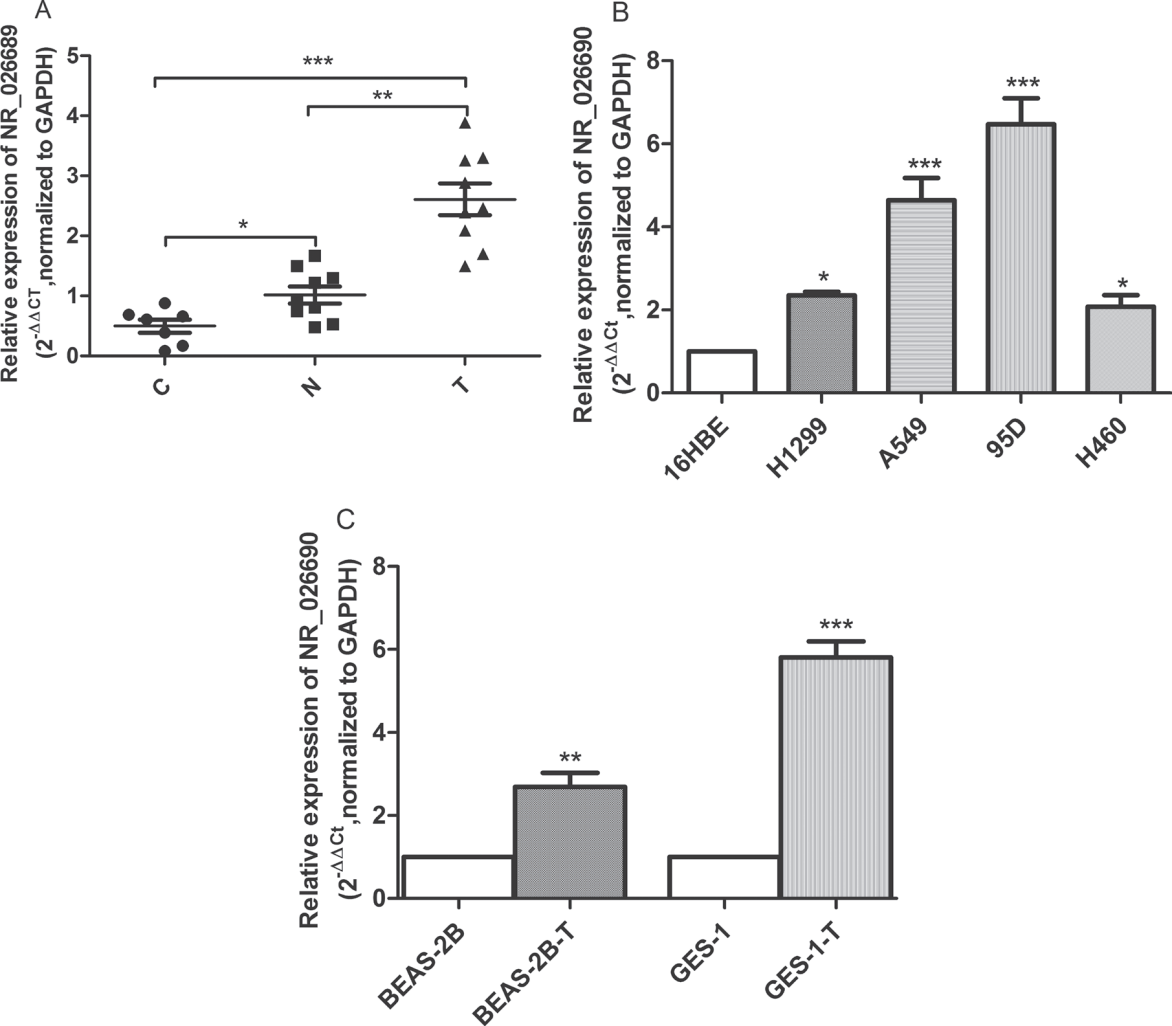
The level of NR_026689 and its human orthologous gene NR_026690 expression Scatter plots of the level of NR_026689 in rat normal lung tissues from control (C, *n* = 7), normal lung tissues in the NNK treatment group (N, *n* = 9), and paired lung tumor tissues from the NNK treatment group (T, *n* = 9) (**A**). The level of NR_026690 in lung cancer cell lines (H1299, A549, 95D and H460) relative to normal human bronchial epithelial cell 16HBE (**B**). The relative level of NR_026690 in benzo (a) pyrene-induced transformation cell (BEAS-2B-T) and MNNG-induced transformation cell (GES-1-T) (**C**). The expression level of NR_026689 and NR_026690 was normalized to GAPDH. *p < 0.05; **p < 0.01, ***p < 0.001.

In order to further explore the association between the alteration in NR_026689 and lung tumor or NNK treatment, in addition to nine paired lung tumor tissues and normal lung tissues from the NNK treatment group, we examined the expression levels of NR_026689 in seven normal rat lung tissues from the control group. The differential expression of NR_026689 among the three groups was analyzed using the Kruskal-Wallis test. As shown in Figure 5A, compared with normal lung tissues from the control group, the level of NR_026689 was significantly increased in normal lung tissues from the NNK treatment group (*p* < 0.05) and lung tumor tissues from the NNK treatment group (*p* < 0.001). Compared with normal lung tissues from the NNK treatment group, NR_026689 expression level was significantly increased in paired lung tumor tissues from the NNK treatment group (*p* < 0.01). These results showed that the alteration in lncRNA NR_026689 was associated with NNK exposure and lung carcinogenesis induced by NNK.

According to NCBI, the orthologous gene of NR_026689 in human is NR_026690. In order to explore the potential clinical significance of lncRNA NR_026689 in human lung cancer cell lines and transformation cell induced by chemicals, we detected the expression level of NR_026690 in normal human bronchial epithelial cell 16HBE and lung cancer cell lines (H1299, A549, 95D and H460). The results showed that the level of NR_026690 was significantly increased in lung cancer cell lines compared to 16HBE (Figure 5B). Meanwhile, we detected the expression level of NR_026690 in normal human bronchial epithelial cell BEAS-2B, benzo (a) pyrene-induced transformation cell (BEAS-2B-T), normal human gastric mucosa epithelial cells GES-1 and MNNG-induced transformation cell (GES-1-T). The results showed that the level of NR_026690 was significantly up-regulated in BEAS-2B-T cell and GES-1-T cell. The results showed that the level of NR_026690 was significantly increased in transformed cell induced by chemicals (Figure 5C).

**Figure 6:**
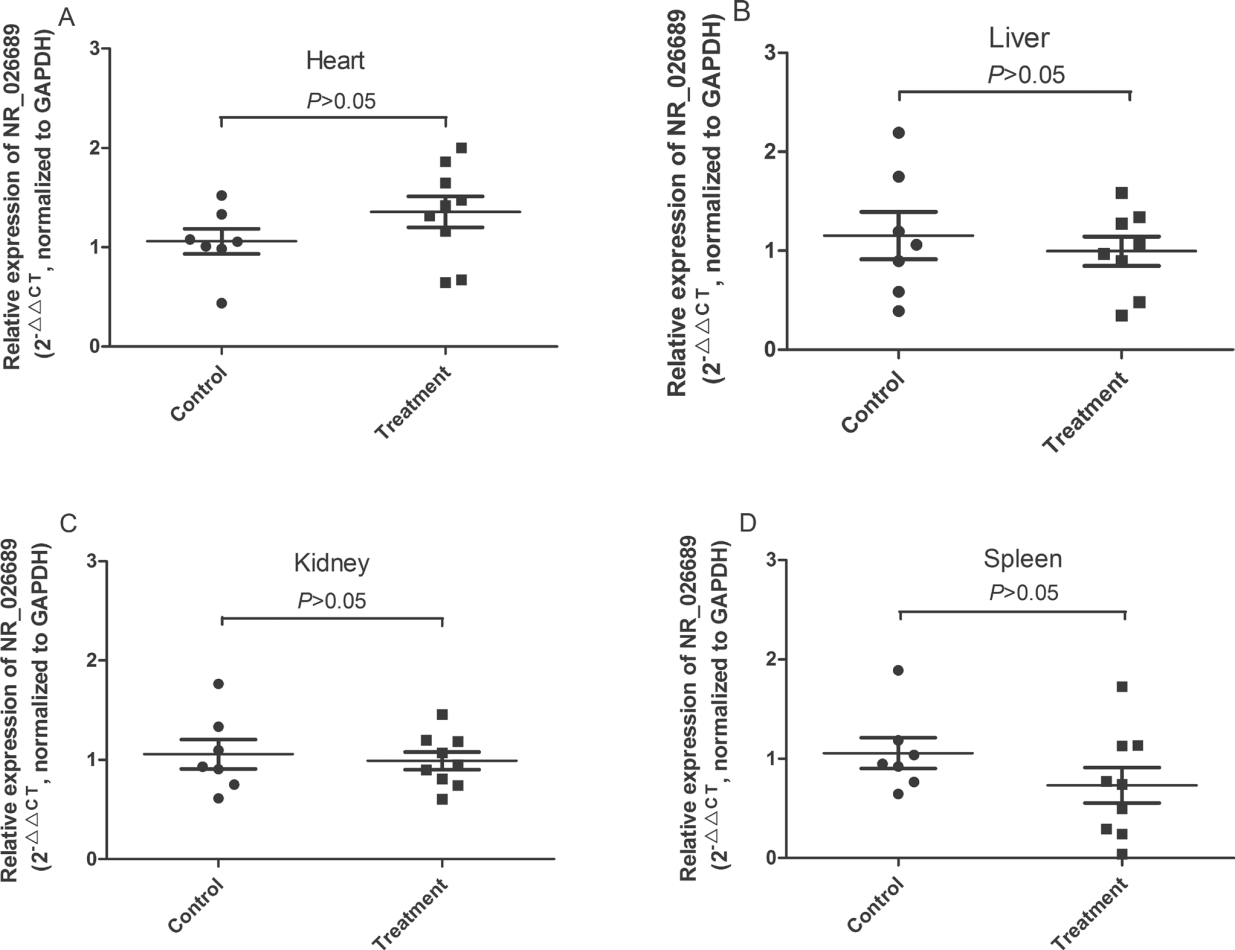
The level of NR_026689 expression in rat other organ tissues from the control group and NNK-treatment groups Levels of NR_026689 expression in the heart (**A**), liver (**B**), kidney (**C**) and spleen (**D**) tissues in rats treated with NNK were determined using qRT–PCR and compared with those of control rats (*n* = 7 for control group; *n* = 9 for NNK treatment group) at the 95th week. Statistical differences were determined by an unpaired Student's *t* test.

In addition, to further analyze the organ specificity in rat of lncRNA NR_026689 expression alteration, the level of NR_026689 was determined in rat heart, liver, spleen and kidney tissues, respectively. As shown in Figure 6, compared with the control group, no significant difference was observed in rat heart (Figure 6A), liver (Figure 6B), kidney (Figure 6C) and spleen (Figure 6D) tissues in the NNK treatment group. These data indicate that the alteration of lncRNA NR_026689 may be specifically observed in lung tissues and associated with lung carcinogenesis induced by NNK.

### Alteration of lncRNA NR_026689 in the blood of rats treated with NNK

**Figure 7:**
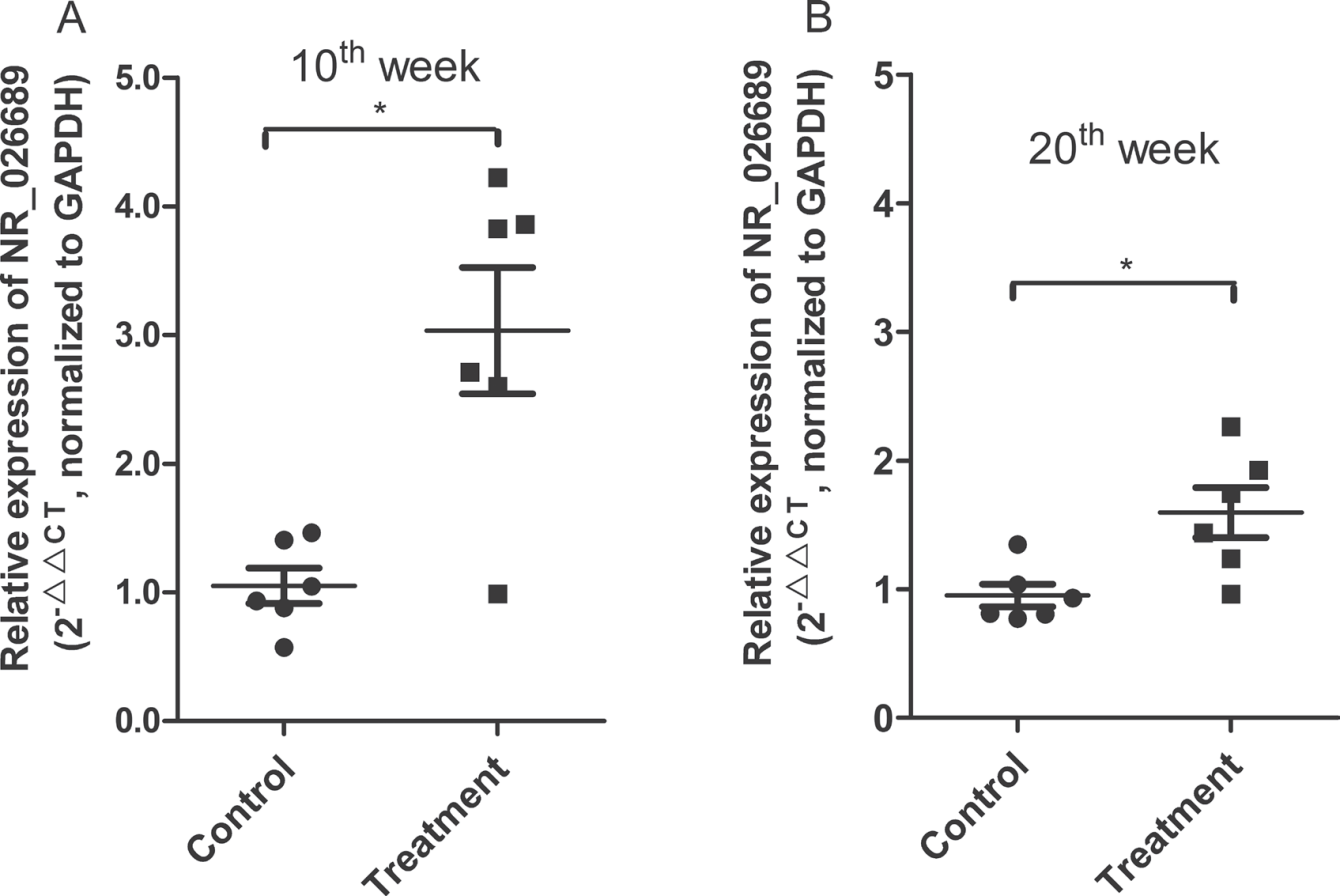
Changes of NR_026689 expression in rat whole blood at early stages following NNK treatment The scatter plot comparing blood NR_026689 levels in individual rats between the control group and the NNK treatment group at weeks 10 (**A**) and 20 (**B**), respectively. GAPDH was used as the reference gene. Each sample was analyzed in triplicate. The Mann-Whitney test was used to determine statistical significance. A single asterisk indicates a significant difference from the control (*p < 0.05).

The lncRNA microarray results of two blood samples indicated that NNK exposure could induce an alteration in lncRNA expression profiles in circulating blood samples. To validate whether blood lncRNA NR_026689 expression is altered and may be a potential biomarker for lung cancer, we investigated the expression change of lncRNA NR_026689 in rat whole blood at early stages of NNK-induced lung carcinogenesis. According to our previous study [27], at the 20th week following NNK treatment, no lung tumors or pathological changes were observed in rat lung tissues in both control and NNK treatment groups. Therefore, we respectively collected whole blood samples from six rats in the control group and six rats in the NNK treatment group at the 10th and 20th week of NNK treatment. Using qRT-PCR, the level of lncRNA NR_026689 was determined in these whole blood samples. As shown in Figure 7, compared with the control group, at the 10th and 20th week, the level of NR_026689 in rat whole blood was significantly up-regulated in the NNK treatment group (Figure 7A and 7B, *p* < 0.05). These results indicated that the level of NR_026689 was significantly altered in rat whole blood from the NNK treatment group vs. the control group during early lung carcinogenesis induced by NNK.

## DISCUSSION

Several recent studies have indicated that environmental carcinogens can induce alterations in lncRNA expression [21, 29]. According to Thai et al., cigarette smoke extract can induce alterations in expression of the smoke and cancer-associated lncRNA-1 (SCAL1) [23]. NNK is a key compound in cigarette smoke and a lung carcinogen with organ-specificity. Silva et al. added NNK to normal human bronchial epithelial cells (NHBE) and identified 12 Long Stress-Induced Non-coding Transcripts [28]. To our knowledge, no study has reported that alterations in lncRNA expression are associated with lung carcinogenesis induced by chemical carcinogens. Using the rat model of lung cancer constructed in our previous study, we determined the lncRNAs associated with lung carcinogenesis induced by NNK.

Recently, many studies have shown that alterations in lncRNA expression profile occur in a variety of human cancers [30]. The lncRNA, HOTAIR, showed increased expression in primary breast tumors and metastases [31]. According to global microarray and *in situ* hybridization (ISH) analyses, Hu et al. identified a novel lncRNA, GAPLINC, which showed increased expression in gastric cancer tissues [12]. Using RNA sequencing, Crea et al. found that lncRNA PCAT18 was specifically expressed in prostate cancer tissues compared to normal tissues [18]. In the present study, to investigate the lncRNAs associated with lung cancer and NNK exposure, the lncRNA differential expression profile was assessed between lung cancer tissues and matched normal lung tissues. Compared with normal lung tissues, the lncRNA expression profile was significantly altered. Similarly, the lncRNA expression profile in blood samples was also changed. These results suggest that lncRNA may be involved in lung carcinogenesis induced by NNK.

According to the lncRNA microarray data and further validation using qRT-PCR, we identified a novel lncRNA NR_026689, which was significantly increased expression in rat lung cancer tissues induced by NNK and is located on chromosome 12q12 in rats. To date, the character and function of NR_026689 are unknown. Using 5′RACE assay, we analyzed the full length sequence and identified the character of NR_026689. The results indicate that NR_026689 is unlikely to encode protein and is an lncRNA. According to Bhan et al., bisphenol-A and diethylstilbestrol exposure can induce alterations in the expression of the breast cancer associated long noncoding RNA, HOTAIR [20]. Female B6C3F1 mice were exposed to the carcinogen, furan, and differentially expressed lncRNAs were observed in liver tissue in the 4.0 mg/kg and 8.0 mg/kg dose groups [22]. lncRNA DQ786227 was identified as an oncogene which showed increased expression and effected cell proliferation and apoptosis in malignantly transformed BEAS-2B cells induced by benzo(a)pyrene [32]. These studies indicated that lncRNA may be involved in carcinogenesis induced by chemical carcinogens. In our present study, compared with normal lung tissues from the control group, the level of NR_026689 was significantly increased in normal lung tissues and lung tumor tissues from the NNK treatment group. These results indicate that the alteration in lncRNA NR_026689 is associated with NNK exposure and lung carcinogenesis induced by NNK. The further results showed that NR_026690, the orthologous gene of NR_026689 in human, was significantly increased expression in human lung cancer cell lines and transformed cell induced by chemicals. According to Yang and Lin et al., the level of lncRNA NR_026690 (also known as ABHD11-AS1) was significantly increased in gastric cancer tissues [33, 34]. These results indicate that lncRNA NR_026689 and its human orthologous gene NR_026690 are associated with cancer and carcinogenesis induced by chemicals.

Numerous studies have shown that lncRNA is a potential and efficient biomarker in the diagnosis and prognosis of cancer. A lncRNA profile study revealed that three-lncRNA signature was associated with the prognosis of patients with esophageal squamous cell carcinoma [35]. Redis et al. identified a novel lncRNA CCAT2 as biomarker for breast cancer [36]. Prensner et al. identified and validated a highly expressed lncRNA, SChLAP1, as a potential biomarker for the prognosis and metastasis of prostate cancer [37]. LncRNA acts as a biomarker for a variety of cancers, and organ specificity of lncRNA expression alteration is crucial. In this study, we found that NR_026689 expression was not significantly different in other major organ tissues in the NNK treatment group compared with the control group. According to Hecht SS, NNK is a potent lung carcinogen in rats and the organ specificity of NNK for the lung is remarkable [25]. Our results indicate that lncRNA NR_026689 may be specifically expression change in lung tissues and a potential biomarker for lung carcinogenesis induced by NNK.

Accumulating evidence shows that circulating lncRNA is a potential and noninvasive biomarker of human diseases including cancer. Using high-throughput methods of RNA analysis, lncRNAs in human plasma were observed and characterized [38]. Tong et al. identified a novel lncRNA, POU3F3, in plasma which is a novel biomarker for the diagnosis of esophageal squamous cell carcinoma [39]. Dong et al. reported that circulating lncRNA CUDR, LSINCT-5 and PTENP1 may be potential biomarkers for distinguishing patients with gastric cancer from healthy controls [40]. Tan et al. reported that circulation lncRNAs could act as biomarkers for predicting tumorigenesis and metastasis in hepatocellular carcinoma [41]. At early stages of mouse liver tumor promotion induced by Phenobarbital, Dlk1-Dio3 ncRNAs were identified as novel candidate early biomarkers for mouse liver tumor promotion [42]. In the present study, we determined the expression of NR_026689 in rat whole blood at early stages of NNK-induced lung carcinogenesis. Compared with the control group, the expression level of NR_026689 in rat whole blood at the 10th and 20th week of NNK treatment was significantly up-regulated in the NNK treatment group. These results indicate that blood lncRNA NR_026689 may be a potential early biomarker for lung carcinogenesis induced by chemical carcinogens.

In conclusion, our findings suggest that lncRNA may be involved in lung carcinogenesis induced by chemical carcinogens. In addition, we identified the novel lncRNA NR_026689. The level of lncRNA NR_026689 was significantly increased in lung tumor tissues induced by NNK and in rat whole blood. These findings suggest that lncRNA NR_026689 may be a potential early biomarker for NNK induced lung cancer and associated with lung carcinogenesis induced by chemical carcinogens.

## MATERIALS AND METHODS

### Collection of rat major organs tissues and blood samples

In our previous study, a NNK-induced rat lung cancer model was constructed [27]. Rat whole blood was collected by orbital bleed at 10 and 20 weeks following NNK treatment. Approximately 1.0 ml whole blood was collected from each rat every time. A volume of 250 μL whole blood was added to a 2 ml microcentrifuge tube with 650 μL RNAlater solution (Ambion, Austin, TX, USA), and mixed thoroughly by inverting the tube several times. The blood samples were first incubated in RNAlater solution overnight at 4°C and then transferred to −20°C until use. 95 weeks after NNK treatment the rats were anesthetized with CO_2_ and sacrificed. Tumor lung tissues and corresponding normal lung tissues were isolated. The pathological type of the lung tumors was moderately differentiated adenocarcinoma. No tumors were found in other organs, such as heart, liver, kidney and spleen tissues. These major organs tissues were also isolated and placed into sterile freezing vials and stored immediately in liquid nitrogen for further use.

### Cell culture and reagents

The lung cancer cell lines (H1299, A549, 95D and H460) were purchased from the Chinese Academy of Sciences Cell Bank of Type Culture Collection (Shanghai, China), and cultured in RPMI-1640 medium (Invitrogen, Carlsbad, CA) supplemented with 10% fetal bovine serum (FBS; Sijiqing Co., Ltd, Hangzhou, China). Normal human bronchial epithelial cells (16HBE), kindly provided by Dr. Xujun (Guangzhou Institute of Respiratory Diseases), and the human gastric epithelial cell line GES-1 obtained from the Cell Resource Center of XiangYa Central Laboratory (Changsha, China), were maintained in minimum essential medium supplemented with 10% FBS (Sijiqing Co., Ltd). BEAS-2B cell kindly provided by Lijin Zhu (Institute of Hygiene, Zhe-jiang Academy of Medical Sciences, Hangzhou, China), were cultured in DMEM medium (Invitrogen, Carlsbad, CA, USA) supplemented with 10% FBS (Sijiqing Co., Ltd). BEAS-2B-T and GES-1-T transformed cells were constructed by our laboratory. All cells were incubated at 37°C in a humidified atmosphere of 5% CO_2_.

### Extraction of RNA

Total RNA in rat organ tissues and cell lines was extracted using Trizol reagent (Invitrogen, USA) according to the manufacturer's instructions. Total RNA in rat whole blood was extracted using the RiboPure^™^ -Blood Kit (Ambion, Austin, TX, USA) according to the manufacturer's instructions. The quality and quantity of the RNAs were assessed by reading at A260/A280 nm using a NanoDrop1000 spectrophotometer (NanoDrop Technologies, Wilmington, DE, USA). RNA integrity was determined by running an aliquot of the RNA samples on a denaturing agarose gel stained with ethidium bromide. The ratio of 28S ribosomal RNA to 18S ribosomal RNA was approximately 2:1, indicating that the RNA samples were intact.

### LncRNA microarray analysis

Three lung tumor tissues and three matched normal lung tissues and two blood samples from the control and NNK treatment group in the 95th week were collected and total RNA was extracted using Trizol reagent. For lncRNA microarray analysis, Arraystar rat lncRNA array (version 1.0, Rockville, Maryland, USA) and an Agilent Array platform were employed. Sample preparation and microarray hybridization were performed based on the manufacturer's standard protocols. Briefly, 1 μg of total RNA from each sample was amplified and transcribed into fluorescent cRNA using Agilent's Quick Amp Labeling protocol (version 5.7, Agilent Technologies). The labeled cRNAs were hybridized onto the Rat lncRNA Array (4 × 44K, ArrayStar). After the slides were washed, the arrays were scanned by an Agilent Scanner G2505B. Acquired array images were analyzed using Agilent Feature Extraction software (version 10.5.1.1, Agilent Technologies). Quantile normalization and subsequent data processing were performed using the GeneSpring GX v11.5 software package (Agilent Technologies, Santa Clara, USA). After normalization and further screening, differentially expressed lncRNAs and mRNAs showing statistical significance were identified by Volcano Plot filtering. Pathway and GO analysis were applied to determine the roles of these differentially expressed mRNAs and hierarchical clustering was performed to show the distinguishable lncRNAs and mRNAs expression patterns among the samples.

### Quantitative reverse-transcription PCR (qRT-PCR)

Expression levels of candidate lncRNAs were determined by qRT-PCR using the PrimeScript^™^ RT Master Mix (perfect Real Time) reagent kit (Takara Bio Inc. Dalian, P.R. China). All reactions were carried out according to the manufacturer's instructions. Briefly, 500 ng total RNA was reverse-transcribed with 5 × PrimeScript RT Master Mix (perfect Real Time). The reverse-transcription reaction was performed according the manufacturer's recommendations (37°C for 15 min, 85°C for 5 s and incubated at 4°C). The expression levels of the lncRNAs were determined with qRT-PCR using the PrimeScript^™^ RT Master Mix (perfect Real Time) reagent kit (RR036A, Takara Bio Inc. Dalian, P.R. China) and the ABI 7500 real-time PCR system (Applied Biosystems). The conditions for the reactions were those recommended by the manufacturer (95°C for 30 s, followed by 40 cycles at 95°C for 5 s and 60°C for 30 s). The cycle threshold (Ct) values were calculated with the SDS 2.0.5 software (Applied Biosystems). The average expression levels of the lncRNAs were normalized to the average for GAPDH using the 2^−ΔΔCt^ method. All qRT-PCR experiments were performed at least three times in triplicate. The primers used for qRT-PCR are listed in Supplementary Table S1.

### 5′RACE assay

Rapid amplification of cDNA ends (RACE) special primers were designed by Primer Premier 5.0 according to sequence information of NR_026689 transcript. The 5′end of NR_026689 was obtained using the BD SMART^™^ RACE cDNA Amplification Kit according to the manufacturer's instructions. Briefly, total RNA was isolated from lung tissues and reversed using the MMLV first Strand cDNA Synthesis Kit (BD Biosciences Clontech, CA, USA) and a PCR purification kit (Sangon, Shanghai, P.R. China). This was followed by the nested PCR with adaptor primer and gene-specific PCR primers. The primers used for nested PCR are shown in Supplementary Table S1.

### Statistical analysis

Statistical analyses were carried out using SPSS statistical analysis software (SPSS Inc., Chicago, IL, USA). For parametric comparisons, the two independent samples *t* test and paired samples *t* test were used, respectively. For nonparametric comparisons, the Mann-Whitney test (for two nonparametric groups) and the Kruskal-Wallis test (for more than two nonparametric groups) were used. All data are presented as mean ± standard error of mean (SEM) for three independent experiments. A *p* value < 0.05 was considered statistically significant.

## SUPPLEMENTARY MATERIAL TABLE


