# Supplementary material for: Identification of a long non-coding RNA NR_026689 associated with lung carcinogenesis induced by NNK
Source: Oncotarget. 2016 Feb 18;7(12):14486–98. doi: 10.18632/oncotarget.7475 (PMC4924730; doi:10.18632/oncotarget.7475)
Supplement: Supplementary file 1 [file oncotarget-07-14486-s001.pdf]

Identification of a long non-coding RNA NR_026689 associated with lung carcinogenesis induced by NNK

Supplementary Materials

Supplementary Table S1: Primer sequence list for qRT-PCR and 5′RACE assay

Gene	Sequence
GAPDH	5′-ACAGCAACAGGGTGGTGGAC-3′
	5′-TTTGAGGGTGCAGCGAACTT-3′
NR_026689	5′-TCATCCATCACCTTCCAACA-3′
	5′-ACCGCTCGCTTCTTAGCAAT-3′
NR_027324	5′-AGCTCGGACTGGAGACTAGG-3′
	5′-CATCACACCGGACCATGTCA-3′
NR_002704	5′-GTGATGGGACATCTGGTGGA-3′
	5′-CCTTGTGTGGAGCTTGCCAT-3′
NR_024118	5′-GACATGCAGACTGACGGAGG-3′
	5′-GCAGGTCCACACGCATAGAG-3′
NR_027235	5′-GTGCCAAGCAGTTTCAGGAG-3′
	5′-TCCCAAGAGGAGAGCAACAC-3′
NR_026690	5′-CTCCACCTGACAGCAACATC-3′
	5′-TTCTTGGCAATGGCTTCA-3′
5′ RACE Outer Primer	5′-GCTGTCAACGATACGCTACGTAAC-3′
5′ RACE GSP1	5′-CGCTTCTTAGCAATGGCTTCATCCCT-3′
5′ RACE inner primer	5′-GCTACGTAACGGCATGACAGTG-3′
5′ RACE GSP2	5′-CCTGCCGCTTAGGATTCTGGGTTCTG-3′
